# Mutation of a *Salmonella* Serogroup-C1-Specific Gene Abrogates O_7_-Antigen Biosynthesis and Triggers NaCl-Dependent Motility Deficiency

**DOI:** 10.1371/journal.pone.0106708

**Published:** 2014-09-11

**Authors:** Xiujuan Zhou, Bin Liu, Chunlei Shi, Xianming Shi

**Affiliations:** 1 MOST-USDA Joint Research Center for Food Safety, School of Agriculture & Biology, and State Key Lab of Microbial Metabolism, Shanghai Jiao Tong University, Shanghai, China; 2 College of Food Science and Engineering, Northwest Agriculture & Forestry University, Shaanxi, Yangling, China; Indian Institute of Science, India

## Abstract

Several molecular detection marker genes specific for a number of individual *Salmonella* serogroups have been recently identified in our lab by comparative genomics for the genotyping of diverse serogroups. To further understand the correlation between serotype and genotype, the function of a *Salmonella* serogroup-C1-specific gene (*SC_2092*) was analyzed in this study. It was indicated from the topological prediction using the deduced amino acid sequence of *SC_2092* that this putative protein was highly similar to the confirmed Wzx flippases. Furthermore, SDS-PAGE revealed that lipopolysaccharide (LPS) biosynthesis, specifically O-antigen synthesis, was incomplete in an *SC_2092* in-frame deletion mutant, and no agglutination reaction with the O_7_ antibody was exhibited in this mutant. Therefore, it was revealed that this *Salmonella* serogroup-C1-specific gene *SC_2092* encoded a putative flippase, which was required for O_7_-polysaccharide biosynthesis, and was designated here as *wzx_C1_*. Subsequently, the effects of the deletion of *wzx_C1_* on bacterial motility and sodium chloride (NaCl) tolerance were evaluated. The *wzx_C1_* mutant lacked swarming motility on solid surfaces and was impaired in swimming motility in soft agar. Moreover, microscopic examination and RT-qPCR exhibited that an increased auto-aggregation and a strong defect in flagella expression, respectively, were responsible for the reduced motility in this mutant. In addition, the *wzx_C1_* mutant was more sensitive than the wild-type strain to NaCl, and auto-aggregation of mutant cells was observed immediately up on the addition of 1% NaCl to the medium. Interestingly, the motility deficiency of the mutant strain, as well as the cell agglomeration and the decrease in flagellar expression, were relieved in a NaCl-free medium. This is the first study to experimentally demonstrate a connection between a *Salmonella* serogroup specific gene identified by comparative genomics with the synthesis of a specific O-antigen biosynthesis. Also, our results show that the mutation of *wzx_C1_* triggers a NaCl-dependent motility deficiency.

## Introduction

The genus *Salmonella* is comprised of a heterogeneous group of Gram-negative bacteria, differentiable by biochemical and serological properties. The O-antigen (the main component of the bacterial cell wall LPS) contributes major antigenic and immunogenic characteristics, and is a basis of *Salmonella* serotype diversity. On the basis of the structural variation in O-antigens, *Salmonella* has been divided into 46 serogroups [1]. The most common O-antigen serogroups are A, B, C1, C2 and D, strains of which cause approximately 70% of *Salmonella* infections in humans and animals [2]–[5]. Each *Salmonella* serogroup is defined by an antigenic formula that indicates the specific O-antigens present in the LPS of strains belonging to that serogroup. For example, *Salmonlla enterica* serovar Choleraesuis (*S.* Choleraesuis), which causes extra-intestinal infections or sepsis in humans [6], [7] and has a higher mortality rate in humans than other *Salmonella* serovars [8], and strains of other sevovars within serogroup C1 have the antigenic formula O:6,7,14, indicating the presence of O_6_^−^ O_7_^−^ and, in some cases, O_14_-antigens. The underline for the O_14_-antigen in the formula indicates that this antigen is only present if the strain is lysogenized by a converting phage. Furthermore, the O_6_^−^ and O_14_^−^antigens are present in the LPS of serogroups other than C1; thus, only the O_7_-antigen is unique to serogroup C1. It has been reported that strains of serogroup C1 represented 20% to 35% of all *Salmonella* isolated from fecal samples of beef and dairy cows in the United States [9]. Also, the latest reports from the United States Centers for Disease Control and Prevention in 2012 showed that 12 acute salmonellosis cases (total 50 cases) were caused by strains from *Salmonella* serogroup C1 [10]. Although many highly virulent serovars are from *Salmonella* serogroup C1, this serogroup has not received sufficient attention from researchers, specifically the synthesis of their unique O-antigen.

Nearly all *E. coli* and *Salmonella* O antigens are synthesized by the Wzx (flippase)/Wzy (polymerase)-dependent pathway [11]. Genes encoding enzymes involved in O-antigen biosynthesis are mostly grouped together on the chromosome in an O-antigen *rfb* gene cluster, and the structural difference of the O-antigens is generally mirrored by genetic distinction in these clusters [12]. *Salmonella* serogroup C1 has a different O-antigen structure than that from serogroups A, B, C2 and D, and genes in the *rfb* region of *Salmonella* serogroup C1 show no significant similarity to that of other *Salmonella* serogroups [13]. Although studies of the O-antigen biosynthesis in *Salmonella* serogroup C1 were initiated several years ago, the understanding of its genetic pathway still needs to be resolved.

In addition, O-antigens have been demonstrated to be involved in diverse interactions between bacterial cells and the environment. Several research groups have recently described an effect on motility in O-antigen mutant strains, however, the explanations for the role of O-antigen on motility behavior are diverse. O-antigen mutants in *Myxococcus xanthus* are defective in S (social) motility [14]; Mutants of *S.* Typhimurium lacking O-antigen synthesis showed normal swimming motility but conditional defects on swarming motility [15]; The deletion of O-antigen in *E. coli* showed significant defects in both swimming and swarming motilities [16]; Loss of O-antigen ligase in *Proteus mirabilis* inhibited swarming motility on solid surfaces [17]. At present, several mechanisms for O-antigen's function in motility have been identified, affecting motility in diverse ways, including relief of RcsB-mediated repression of flagellin gene expression [16], [17] and reducing surface friction by acting as a wettability agent [15]. However, it is thought that there are additional unknown roles for O-antigen involved. Therefore, further studies are needed to achieve a more comprehensive understanding of the role of O-antigen in bacterial motility.

Recently in our lab, we found seven genes that were conserved and specific for *Salmonella* serogroup C1 by comparative genomic analysis [18]. According to preliminary analyses, most of the C1-specific genes encode membrane proteins with high numbers of transmembrane segments (TMs). One conserved *Salmonella* serogroup C1-specific gene (locus SC_2092 in *S.* Choleraesuis; GenBank Accession # NC_006905) that putatively encodes a protein with 12 TMs is within the O_7_ antigen *rfb* gene cluster [19] and may be involved in translocation of O-antigens across the cytoplasmic membrane. Although this O-antigen processing protein shares very little amino acid sequence similarity with known Wzx flippases, it's membrane topology is similar to other *wzx* gene products extensively studied in *Pseudomonas aeruginosa* PAO1 [20], *E. coli* O157:H7 [21] and *S. enterica* serovar Typhimurium [22], with high numbers of predicted α-helical TMs. Herein this *Salmonella* serogroup C1-specific gene is designated *wzx_C1_*. Currently there is no precise explanation for the high level sequence variation among *wzx*/Wzx genes/proteins, however, this sequence diversity may be related to the diversity of O-unit structures.

The aim of this study was to determine whether the putative *wzx_C1_* gene could affect the synthesis of *Salmonella* O-antigen. Using *S.* Choleraesuis ATCC 10708 as a representative strain of *Salmonella* serogroup C1, we employed a combination of bioinformatics as well as mutagenesis and LPS composition analysis, to provide experimental evidence that the *wzx_C1_* gene specific for *Salmonella* serogroup C1 encodes an enzyme involved in LPS biosynthesis, specifically required for synthesis of the unique O_7_ antigen. We demonstrate further that this O_7_ antigen deletion affects *Salmonella* motility and its responses to the environment, including NaCl tolerance and cell-to-cell interactions. These findings will allow a better understanding of the role of this Wzx_C1_ in *Salmonella* serogroup C1, and help us to evaluate its effects on bacterial structure and physiology.

## Materials and Methods

### Bacterial strains and growth conditions

Strains and plasmids used in this work are listed in Table 1. Wild-type *S.* Choleraesuis ATCC10708 (type strain) belonging to *Salmonella* serogroup C1 was obtained from the Shanghai Entry-Exit Inspection and Quarantine Bureau of China. The *SC_2092* deletion mutant △wzx_C1_ and its complemented strain △wzx_C1_-C were generated in this study (Table 1). *S.* Choleraesuis ATCC10708, △wzx_C1_, △wzx_C1_-C and all the *E. coli* strains were cultured in Luria-Bertani (LB) medium (Oxoid, Cambridge, United Kingdom). When necessary, chloramphenicol and ampicillin were added at 35 µg/ml and 100 µg/ml, respectively. The incubation temperature was 37°C and all broth cultures were aerated by shaking at 180 rpm.

**Table 1:** Strains and plasmids used in this study.

Strain or plasmid	Comment(s)	Source/reference
*S.* Choleraesuis ATCC10708	Wild-type, *Salmonella* serogroup C1, Type strain	Shanghai Entry-Exit Inspection and Quarantine Bureau of China
△wzx_C1_	*SC_2092* deletion mutant of *S.* Choleraesuis ATCC10708 by pRE112Δwzx	This study
△wzx_C1_-C	*wzx* deletion mutant complemented with plasmid pREΔwzx-C	This study
*E. coli* TG1	*sup*E, *hsd*Δ5, *thi*Δ(lac-proAB), F′[*tra*D36, *pro*AB+, *lac*Iq, *lac*ZΔM15]	Laboratory stock
*E. coli* SM10 (*λpir*)	*thi thr*-1 *leu*6 *pro*A2 *his*-4 *arg* E2 *lac*Y1 *galK*2, *ara*14*xy*l5 *supE*44, *λpir*	[25]
pMD_18_-T	Cloning vector, Amp^r^	TaKaRa, Japan
pRE112	pGP704 suicide plasmid, *pir* dependent, *ori*T, *ori*V, *sac*B, Cm^r^	[24]
pREΔwzx	pRE112 containing a 1251 bp *wzx*-deletion PCR product (593 bp of sequence upstream and 658 bp of sequence downstream of *wzx_C1_* generated by overlap extension PCR); used to generate strain △wzxC1	This study
pREΔwzx-C	pRE112 containing a wild-type copy *wzx_C1_* and its two flanks sequence; used to complement strain △wzxC1	This study

### Generation of an in-frame *wzx* deletion mutant of *S.* Choleraesuis ATCC10708

PCR-amplified DNA fragments used in the construction of an in-frame *wzx* deletion mutation were generated by overlap extension PCR [23]. Two PCR fragments were obtained from *S.* Choleraesuis ATCC10708 genomic DNA with the primer pairs of wzx_C1_-for (5′-GC**TCTAGA**CCACCAGTTCATAGGCATCT-3′) plus wzx_C1_-int-rev (5′-GAAAGTAAAA GTTAACCAAGCGGGAAAATA-3′) and wzx_C1_-rev (5′-CG**AGCTC C**TATTTTCGTGATTTTGCCT-3′) plus wzx_C1_-int-for (5′- CTTGGTTAAC TTTTACTTTCCGGATGTAAAC-3′) using KOD Dash DNA polymerase (Toyobo, Japan). The products generated by the first PCR were a 593-bp fragment containing the DNA sequence upstream of *wzx* and a 658-bp fragment containing the DNA sequence downstream of *wzx*. The 20-bp overlap sequences (underlined) allowed amplification of a 1251-bp product after a second PCR with the template of the mixture of two first PCR products and primers wzx_C1_-for and wzx_C1_-rev, which introduced *Xba* I and *Sac* I restriction endonuclease sites (bold), respectively. The second PCR product containing a deletion of *wzx* was cloned into the pMD_18_-T vector (TaKaRa, Japan) to generate pMD_18_△wzx, and DNA sequencing was carried out to confirm the correct construction.

The marker-free in-frame deletion mutant of *wzx* in *S.* Choleraesuis was screened by double selection in two steps. In the first step, the pMD_18_△wzx plasmid with the deletion of *wzx* was excised with *Xba* I and *Sac* I, ligated into suicide vector pRE112 [24], which carries a chloramphenicol resistance gene and a sucrose-sensitivity gene *sacB*. The resulting plasmid pRE△wzx was transformed by electroporation (2500 V, 5 ms) into *E. coli* SM10λpir [25]. Then, 1 ml of *E. coli* SM10λpir cells (ca.10^6^ CFU/ml) containing the plasmid pRE△wzx and 3 ml of the wild-type *Salmonella* cells (ca.10^6^ CFU/ml) were mixed in a culture flask (quantities to 10-ml) for 8 h at 37°C to accomplish the conjugation process. Recipient cells were plated on LA (LB with 1.5% agar) supplemented with chloramphenicol (35 µg/ml) to select the trans-conjugant strain SC + pRE△wzx, containing the plasmid integrated into the *S.* Choleraesuis genome as single crossover. In the second step, a colony of SC + pRE△wzx was grown in LB to allow for a second crossover. After overnight growth on LB at 37°C, the SC + pRE△wzx culture was plated on LA containing 8% (w/v) sucrose, which selected for loss of the pRE112 vector (carrying *sacB* gene). Colonies that grew on this medium were tested for chloramphenicol sensitivity to ensure the loss of plasmid by using LA containing chloramphenicol. The resulting strain, *S.* Choleraesuis △wzx_C1_, was confirmed by PCR with primers wzx_C1_-for and wzx_C1_-rev, and sequencing of the resulting PCR-product using the same primers.

### Slide agglutination test, LPS extraction and SDS-PAGE analysis of O-antigen production

The slide agglutination tests were performed using antisera (Ningbo Tianrun Bio-Pharmaceutical Co. Ltd., Zhejiang, China) on the basis of somatic O_7_ antigen according to the Kaufmann-White scheme. Test *Salmonella* in a drop of saline was placed on a slide, and a drop of antiserum was added and mixed. Then the slide was rocked gently for approx. 1 min.

An LPS Extraction kit (iNtRON Biotechnology, Korea) was used following the manufacturer's instruction. LPS was then separated on Ready Gel Precast Tris-HCl polyacrylamide gels with 15% and 5% acrylamide in the separating and stacking gels, respectively (Bio-Rad Laboratories, USA) in buffer with 2% SDS, and fixed overnight in buffer with 10% acetic acid and 40% methanol. The gels were stained with a silver stain kit (Bio-Rad Laboratories, USA) following the manufacturer's instruction. All the solutions were prepared fresh before use. The experiment was repeated three times.

### Topology prediction of this putative transmembrane protein

Topology models were generated by the consensus web server TOPCONS [26] (http://topcons.cbr.su.se/). Five TOPCONS algorithms (SCAMPI-seq, SCAMPI-msa, PRODIV, PRO and OCTOPUS) were used for the prediction of transmembrane helices. In addition, ZPRED was used to predict the Z-coordinate (the distance to the membrane center) of each amino acid, and △G prediction was used to calculate the free energy for membrane insertion of potential TMs. Given the amino-acid sequence of the test protein, the server provided the predicted topology. The prediction took 10–30 s, and the results were displayed graphically and downloaded in text format.

### Motility assays

The bacterial swimming and swarming motility were assayed on media containing different concentrations of agar. With low agar concentrations (0.3%), the bacteria swim through water channels inside the media. With higher agar concentrations (0.5–0.7%), the bacteria swarm over the agar surface.

Swimming and swarming assays were performed as described previously [27]. Moreover, swimming and swarming assays were tested in both standard LA (containing 10 g NaCl per liter) and NA (LA without NaCl) media containing different concentrations of agar. Using sterile toothpicks, single colonies from streak plates were stabbed into the swimming plates (containing 0.3% agar) and incubated for 24 h. At least six independent colonies were checked for each strain tested. For swarming motility assays, 5 µl of an overnight culture grown in LB was spotted directly on each plate (containing 0.6% agar) and allowed to dry for 10 min with the lid removed. Plates were then covered and incubated at 37°C for 24 h before observation. The effect of bio-surfactin on swarming motility was also tested. Briefly, 5 µl of solution containing *Bacillus subtilis* surfactin (10 µg/ml, Sigma-Aldrich, USA) were spotted in the center of the swarming motility assay plate (LA media containing 0.6% agar), allowed to dry for 5 min, inoculated with the test strains on top of the surfactin spot, again allowed to dry for 10 minutes and then incubated for 24 h at 37°C.

### Flagella and bacterial morphological features examined by transmission electron microscopy (TEM)

The morphological features of the mutant and the wild-type strains were examined by transmission electron microscopy (TEM). Samples were prepared for TEM by two different methods. In the first method a suspensions of the *Salmonella* strain was placed on copper grids, allowed to form a film on the grid for two minutes before the excess solution was removed using absorbent paper, and the grids were dried at room temperature [28]. For the second method, thin sections of the *Salmonella* strains were prepared for TEM as previously described [29]. Briefly, fixed bacteria cells embedded in agar were post-fixed using 1% OsO_4_ in 0.05 M sodium cacodylate buffer for 1 h at room temperature. The cells were washed at least three times using sterile water and subsequently dehydrated in an increasing series of 15-minute ethanol rinses (30%, 50%, 70%, and 100%). The dehydrated cells were embedded in Embed-812 resin and sectioned to a thickness of 70 nm. The sectioned blocks were placed on carbon-coated 200 mesh copper grids, and stained with uranyl acetate for 10 min and Sato's lead for 5 min. All samples were observed with a Tecnai G2 spirit Biotwin microscope (FEI, Japan) operated at an accelerating voltage of 120 kV.

### NaCl tolerance measurements and auto-aggregation assay

In order to determine differences in osmotolerance between the wild-type and mutant strains, cultures were grown in NB medium (LB medium without NaCl) with various salt concentrations (from 0% to 9%). Briefly, 50 µl of overnight culture in NB medium were inoculated into each 5-ml culture (NB medium containing diverse salt concentrations) in a 15-mL flask and incubated at 37°C shaking at 180 rpm for 24 h. The OD_600_ was measured and photographs were taken by light microscopy (LM) at the same time.

The auto-aggregation assay was performed based on the method previously described by Shanks *et al.* [30] with slight modifications. Briefly, *Salmonella* cultures were statically grown in 5 ml LB medium at 37°C for 24 h in 16×150 mm test tubes. The upper 0.5 ml was carefully removed to measure its optical density (OD_600_) (recorded as OD_600__prevortex_). The remaining culture in the test tube was then mixed by vortexing to re-suspend the aggregated cells, and 0.5 ml of the suspension was removed and its OD_600_ was measured (recorded as OD600 _postvortex_). The “percent aggregation” was calculated using the formula: 100% * (OD_600 postvortex_ - OD_600 prevortex_)/OD_600 postvortex_.

### RNA preparation and RT-qPCR analysis of gene expression

Wild-type and mutant *Salmonella* cells were grown in LB or NB medium to an OD_600_ around 1.2 for stationary phase samples. One milliliter of the cell culture was harvested by centrifuging at 12000×g for 2 min. The cell pellet was re-suspended in 100 µl TE buffer containing 10 µg/ml of lysozyme (Roche Diagnostics, Penzburg, Germany), incubated at 37°C for 10 min, and the treated cells were once again harvested by centrifuging at 12000×g for 1 min. The extraction of total RNA was performed with TRIzol reagent according to the manufacturer's protocol (Invitrogen, Carlsbad, USA). DNase I treatment and reverse transcription (RT) were performed using the PrimeScript RT reagent kit with gDNA Eraser (TaKaRa, Japan).

Reverse transcription quantitative real-time PCR (RT-qPCR) assays were performed using an iQ Cycler (Bio-Rad, Watford, UK). The primers used are listed in Table 2 and were synthesized by Sangon Co. Ltd. (Shanghai, China). The reaction solution was as follows: ≤1 µg Power SYBR Green PCR Master Mix (2×) (TaKaRa, Japan), 1 µM Forward Primer, 1 µM Reverse Primer, and 1–100 ng cDNA template, and nuclease-free water to bring the solution to 25 µl. The amplification program was as follows: one cycle at 95°C for 4 min, 40 cycles at 95°C for 15 s, 58°C for 15 s and 68°C for 30 s. A melting-curve between 58°C and 95°C was analyzed to check the specificity of the amplification product after each PCR. The *recA* gene was used as a reference. All the samples, including no-template control, were analyzed in triplicate. The reaction efficiency (E) was calculated using the following equation: E =  [10^(1/-s)−1^]×100. Results were analyzed using the comparative critical threshold method (2^−⊿⊿CT^). Gene expression is generally regarded as up- or down-regulation as its relative expression (RE) level is increased or decreased by at least 2-fold, respectively, as previously described by Desroche *et al*. [31] The RE for each gene was measured in triplicate. RE was significantly different when *p*-value was <0.05 using the T-test.

**Table 2:** Primers used for RT-qPCR analysis.

Target mRNA	Gene product function	Sequences (5′–3′)
*flgA*	flagellar basal body P-ring biosynthesis protein FlgA	F:CTGGCTTCAGCGACGAGGTG
		R:GCGGCAACGGCGACATAA
*motA*	forms the ion channels that couple flagellar rotation to proton/sodium	F:CGCATAGCCGTTCAGATT
		R:TGGATTCATTTCACCGTTAG
*motB*	motive force forms the ion channels that couple flagellar rotation to proton/sodium	F:CCCTGCTGTTGGGTGTAA
		R:TTTCTGGTGATGTGGCTGAT
*cheA*	sensory histitine protein kinase, transduces signal between chemo- signal receptors and CheB	F:CCTACTTCATCGTCGGTCAT
		R:GTTTCCCGCGTCTGGTAC
*cheB*	methyl esterase, response regulator for chemotaxis (cheA sensor)	F:GCATCTGCTGGCTTACCTG
		R:TTGCCGAGCGTCTGAATA
*recA*	DNA strand exchange and recombination protein	F:GATTGGCGTGATGTTCGG
		R:GTTTCGCTACCCACGACATT

### Complementation of the *wzx* mutation

To demonstrate that the *wzx* mutation alone was responsible for the observed phenotypes, a complemented strain was generated. Briefly, a 2778-bp DNA fragment containing an intact open reading frame (ORF) of *wzx*, including 593-bp sequence upstream of the translation start site and a 658-bp sequence downstream from the translation stop site, was amplified from the genomic DNA of *S.* Choleraesuis ATCC10708 using the primers wzx_C1_-for and wzx_C1_-rev. The PCR product was cloned into pMD_18_-T (TaKaRa, Japan) to generate pMD_18_△wzx-C, and DNA sequencing was carried out to confirm the correct construction. The 2778 bp DNA fragment containing *wzx* was excised from pMD_18_△wzx-C with *Xba* I and *Sac* I and ligated into suicide vector pRE112. The resulting construct, pRE△wzx-C, was conjugally transferred from *E. coli* SM10λpir into the *S.* Choleraesuis mutant strain △wzx_C1_. The△wzx_C1_ complemented strain (△wzx_C1_-C) was then selected in two steps as described above. The resulting complemented strain, *S.* Choleraesuis △wzx_C1_-C, was confirmed by PCR with primers wzx_C1_-for and wzx_C1_-rev, and DNA sequencing of the resulting PCR-product using the same primers.

## Results

### The *SC_2092* gene encodes a putative flippase Wzx required for O_7_-polysaccharide biosynthesis

The *S.* Cholareasuis gene *SC_2092* is unique to strains of serogroup C1. The gene is located among a number of genes (O-antigen gene cluster) whose products appear to be associated with O_7_-antigen biosynthesis. The deduced amino acid sequence of the protein encoded by gene *SC_2092* has 12 possible transmembrane segments distributed throughout its length, which is the most characteristic feature common to all Wzx proteins that function in transporting the pre-formed O-antigen units to the outside of the cell. Although gene *SC_2092* and its deduced amino acid sequence share little sequence similarity with the *wzx/*Wzx genes/proteins of *S.* Typhimurium (0.8% and 23%, respectively) and *E. coli* O157:H7 (4% and 24%, respectively), the topology prediction profile of the putative Wzx ortholog (the *SC_2092* gene product) from *S.* Choleraesuis was highly similar to Wzx proteins from *S.* Typhimurium and *E. coli* O157:H7 (Fig. S1). All of the programs predicted that the N and C termini of the three above-mentioned proteins were located in the cytoplasm. The number of TMs and the orientation of the intervening loops for the three Wzx proteins predicted by different algorithms gave similar results. Based on these results we propose that the *SC_2092* is a *wzx* gene encoding an O_7_-specific flippase required for O-antigen in strains of *Salmonella* of serogroup C1.

To provide experimental evidence that this putative *wzx* gene plays a role in LPS biosynthesis, we constructed a marker-free *wzx* deletion mutant of *S.* Choleraesuis ATCC10708, and a complemented strain using a double cross strategy. The strain constructs were confirmed by PCR. The results of DNA sequencing for these PCR products further verified the correct deletion and complementation of the allelic exchange mutagenesis (data not shown).

**Figure 1:**
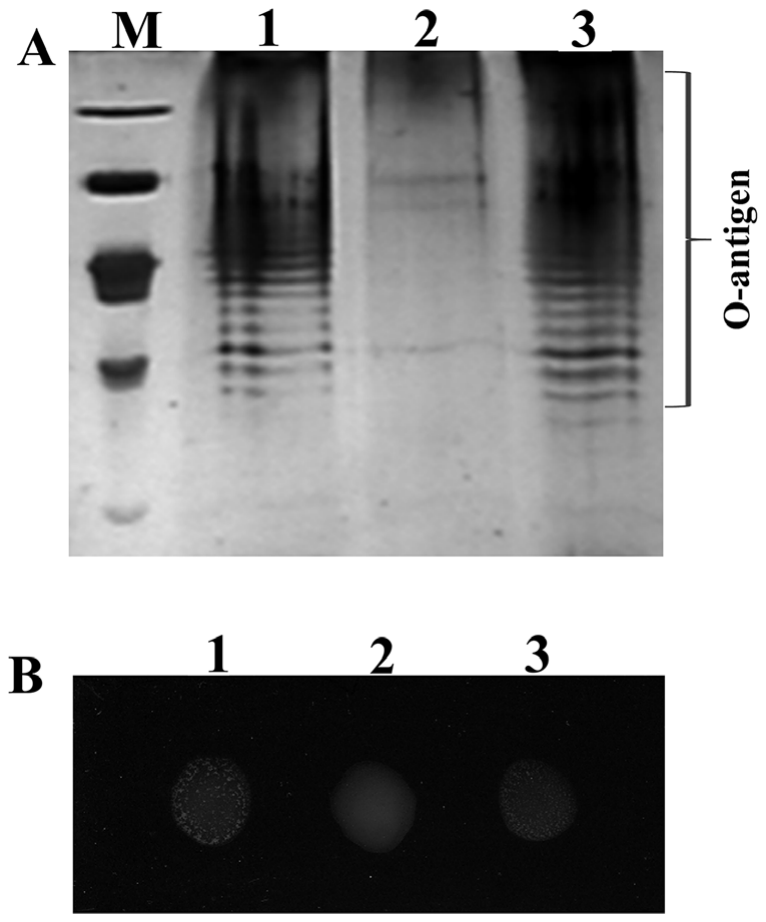
The O_7_-antigen synthesis was blocked in the △wzx deletion mutant. A) The presence of O-antigen was determined by SDS-PAGE separation of LPS preparations followed by silver staining. Lane 1, wild-type *S.* Choleraesuis strain ATCC10708; Lane 2, △wzx mutant strain; Lane 3, the △wzx complemented strain. The O-antigen latter is indicated. B) Agglutination was examined with somatic O_7_ antiserum and pictures were taken within 5 min. Spot 1, wild-type *S.* Choleraesuis strain ATCC10708; Spot 2, the △wzx mutant strain; Spot 3, the △wzx complemented strain.

LPSs of the wild-type, *wzx_C1_* mutant and the complemented strains were isolated and separated by sodium dodecylsulphate polyacrylamide gel electrophoresis (SDS-PAGE) (Fig. 1A). The LPS from the wild-type and the complemented strain exhibited a ladder pattern that is characteristic of LPS with a different number of O-repeat units in the O-antigen, and the upper ladder bands represent this portion of LPS containing O-antigen. In contrast, in the LPS profile of the mutant, the upper bands were completely absent. However, the other lower bands representing the LPS core showed no apparent changes (data not shown), suggesting that LPS O-antigen synthesis was defective in the *wzx* mutant strain. This result was confirmed by the slide agglutination test with somatic O_7_ antiserum (Fig. 1B). Agglutination was obtained in both wild-type and complemented strains; moreover, the positive reactions appeared within 5 to 10 s. However, the negative reactions associated with mutant persisted even when slides were observed for an additional 5 to 10 min. Based on these data, this putative *wzx* gene is designated as *wzx_C1_*, which encodes a putative flippase that is required for O_7_-polysaccharide export in strains of *Salmonella* of serogroup C1.

### The *wzx_C1_* deletion resulted in conditional defects on both swimming and swarming motility

**Figure 2:**
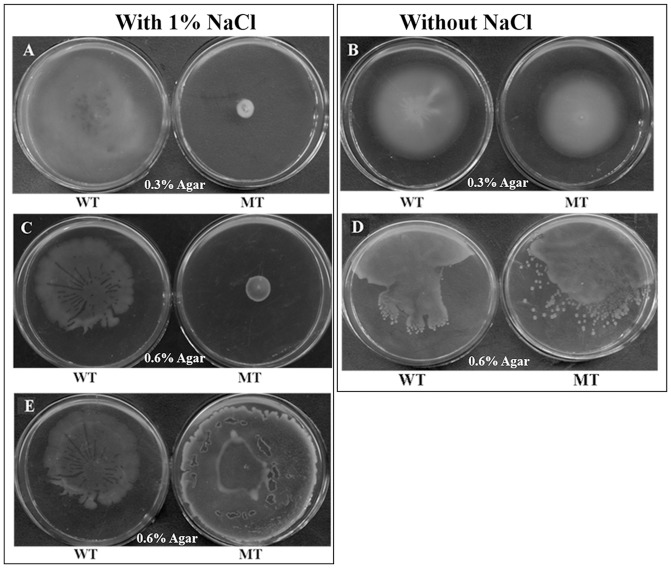
Motility assays of wild-type and △wzx_C1_ mutant strains of *S.* Choleraesuis. Swimming motility of wild-type (WT) and its △wzx_C1_ derivative (MT) were monitored in 0.3% LA plates with 1% NaCl (A) or without NaCl (B). For swarming assays, 0.6% agar plates with 1% NaCl (C) or without NaCl (D) were used. The effect of biosurfactant (surfactin from *B. subtilis*, Sigma-Aldrich, USA) on the swarming phenotype was also tested (E).

The swimming motility of the wild-type and △wzx_C1_ mutant strains were determined using a conventional soft agar test (LB containing 0.3% agar). Twenty-four hours after inoculation, the wild-type strain had produced an area of growth that completely filled the plate, indicating a high degree of swimming motility. However, the growth of the △wzx_C1_ mutant strain had not spread far from the inoculation point, indicating that the strain had reduced motility relative to the wild-type strain. (Fig. 2A). In other words, an in-frame deletion in *wzx* in *S.* Choleraesuis strain △wzx_C1_ significantly reduced swimming motility. A similar result was observed when swarming motility was tested on LB plates containing 0.6% agar (Fig. 2C). Interestingly, both the swimming and swarming motility defects of the △wzx_C1_ mutant strain were rescued in the absence of NaCl (Figs. 2B and 2D). In addition, to test if the hydrophilic O-antigen provides a surfactant function during swarming, the mutant strain was inoculated on swarming medium with a bio-surfactant from *B. subtilis.* The swarming motility of the mutant was recovered by the addition of bio-surfactant (Fig. 2C MT and Fig. 2E MT). The addition of bio-surfactant had little effect on swimming motility (data not shown).

### The mutant had reduced osmotolerance to sodium chloride, and increased auto-aggregation

**Figure 3:**
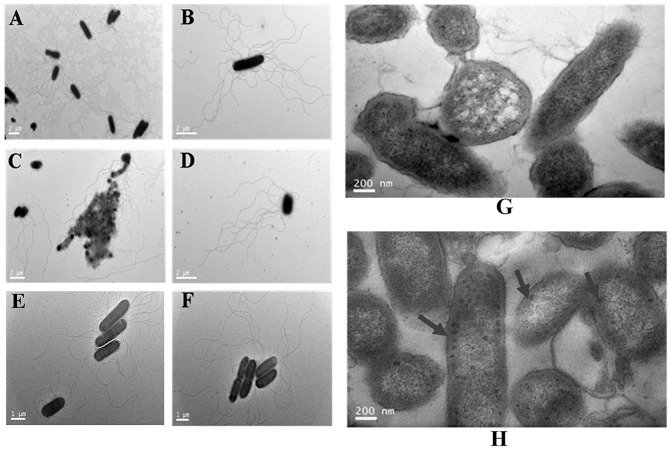
Morphological features in the wild-type and *wzx_C1_* deletion mutant strains revealed by TEM. TEM images of intact cells of wild-type *S.* Choleraesuis strain ATCC10708 (A and B) and the △wzx**_C1_** deletion strain grown in medium with NaCl (C and D) or in NaCl-free medium (E and F). TEM thin sections of wild-type *S.* Choleraesuis strain ATCC10708 (G) and △wzx**_C1_** deletion strain (H). The unknown intracellular crystals in the mutant cells were marked with the arrows.

To determine whether the mutation of *wzx_C1_* affected the morphological features of the mutant strain, transmission electron microscopy (TEM) images of intact wild-type and the mutant cells were collected (Fig. 3A∼F). Relative to the wild-type cells, cells of the *wzx_C1_* mutant formed multicellular aggregates (Figs. 3A and 3C). Nevertheless, the aggregation was alleviated when NaCl was removed from the medium (Figs. 3E and 3F). In addition, TEM of thin sections showed that the mutant strain had no remarkable difference with the wild-type strain in the morphology of bacterial cells and the integrity of cell wall (membrane) (Figs. 3G and 3H). However, unknown intracellular crystals (marked with arrow in Fig. 3H) around the inner membrane were present in most of the mutant cells.

**Figure 4:**
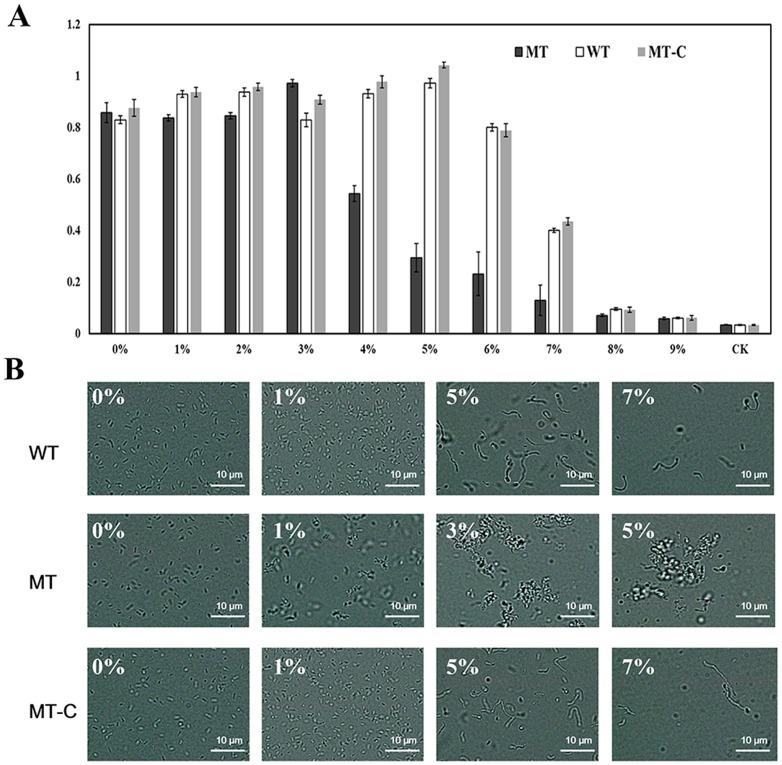
The effect of NaCl concentration on final culture density and cell morphology. The wild-type (WT), △wzx_C1_ mutant (MT) and complemented mutant (MT-C) strains were grown in various concentrations of NaCl for 24 h. (A) The final OD_600_ for cultures grown in different NaCl concentrations (0%–9%) was measured and (B) Morphologies at selected NaCl concentrations were recorded by light microscopy. CK: NB medium as a negative control. The data in (A) are from triplicate samples and images in (B) are representative of the three replicate samples. White bar  = 10 µm.

**Figure 5:**
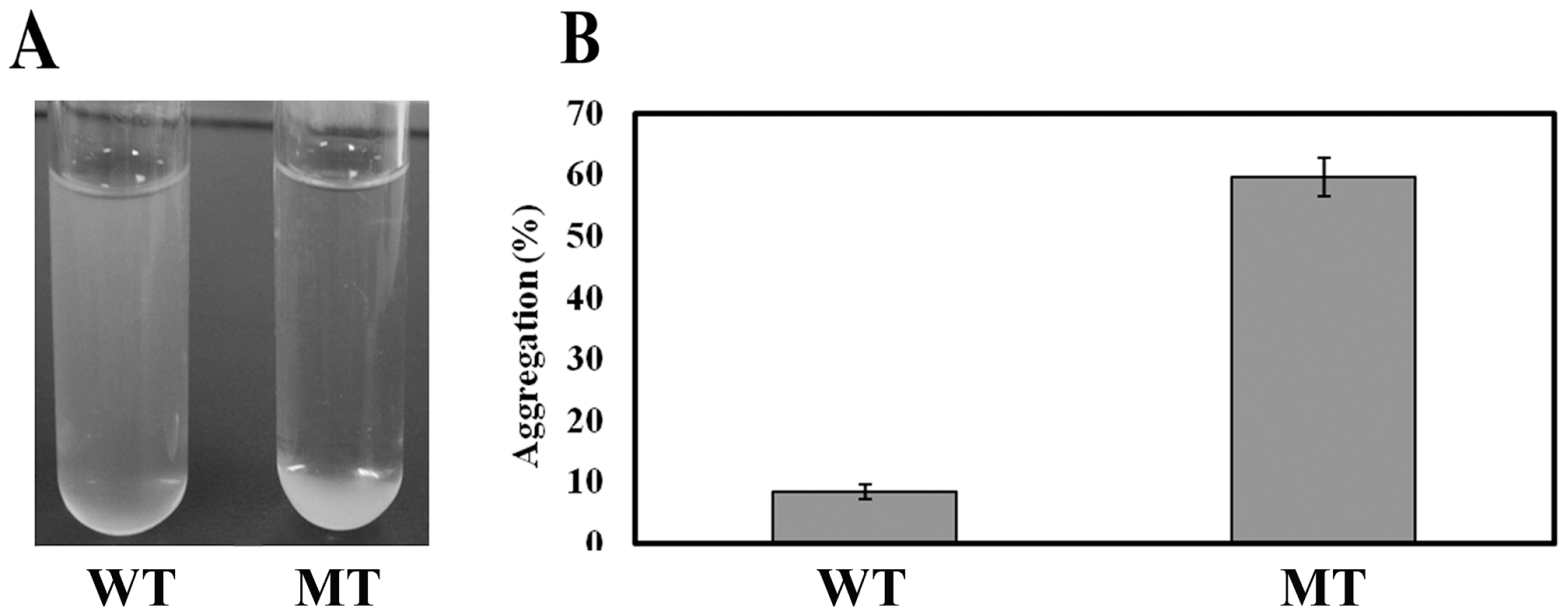
Auto-aggregation of the wild-type and the △wzx_C1_ mutant. Visual aggregation (A) on and percent aggregation (B) of wild-type (WT) and △wzx_C1_ mutant (MT) cultures grown statically for 24 h at 37°C.

The tolerance of the mutant and wild-type strains to NaCl, an osmotic agent, was evaluated, and results showed that the mutant was more susceptible to NaCl. It was observed that while the wild-type and the complemented strain were able to tolerate salt levels as high as 7%, the mutant was sensitive to salt concentrations above 4% (Fig. 4A). Also, cell aggregation, which did not occur in the wild-type or △wzx_C1_-C complemented strain at NaCl concentrations as high as 7%, occurred in the △wzx_C1_ mutant at all salt concentrations tested (as low as 1% NaCl) (Fig. 4B). The auto-aggregation was tested with a culture medium containing 1% NaCl (Fig. 5A) and the “percent aggregation” was calculated using OD_600_ measurements from these cultures. While the wild-type showed 8.37% aggregation, the mutant demonstrated 59.62% aggregation (Fig. 5B). A swimming video recorded by light microscopy (LM) was consistent with conditional defects in motility and aggregation of the mutant strain (data not shown).

### The Wzx_C1_ mutation showed a defect in flagellar gene expression

To distinguish whether the above-observed defect in motility was due to a deficiency in flagellar expression or a restriction in flagellar motion, we first examined flagellar formation by flagella silver staining (data not shown) and TEM observation of the wild-type and the △wzx_C1_ mutant cells. These TEM observations showed that the mutant strain appeared to exhibit reduced flagellation (Fig. 3D), while the wild-type strain had intact peritrichous flagella (Fig. 3B). In addition, the aggregate of mutant cells appears even more diminished of flagella (Fig. 3C).

**Figure 6:**
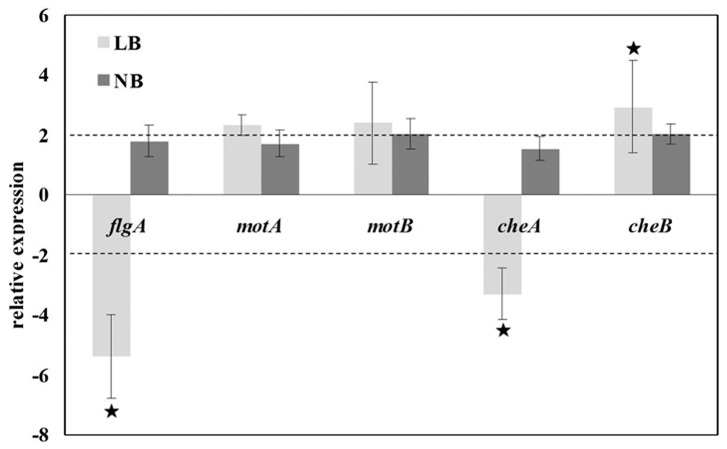
Relative expression of selected swimming motility related genes. Total RNA was extracted from the wild-type and △wzx_C1_ mutant grown in LB and NB medium. *flgA, motA, motB, cheA* and *cheB* expression was analyzed by quantitative RT-PCR. Average fold changes in gene expression in the mutant at each culture condition compared with those of the wild-type are shown. *recA* was used as an internal control. Errol bars indicate standard deviations. Stars indicate that the fold-changes in gene expression were significantly different (*p*-value, 0.05).

To further investigate the nature of the observed decrease in motility and flagellation, the expression levels of five genes (*flgA, motA, motB, cheA* and *cheB*) required for flagella-mediated swimming motility and chemotaxis were determined by RT-qPCR for the wild-type and the △wzx_C1_ mutant cells grown in LB or NB medium (Fig. 6). Compared to the wild-type, the *flgA* gene expression was down-regulated by 5.3-fold in the mutant when the cultures were grown in LB (1% NaCl). In addition, the expression of two chemotaxis genes tested was effected differently (*cheA* decreased and *cheB* increased) in the △wzx_C1_ mutant relative to the wild-type when they were grown in the medium containing NaCl. However, the wild-type and mutant strains examined under a light microscope did not show detectable chemotaxis defect (biased running or tumbling behavior) (data not shown). Nevertheless, the expression of *motA* and *motB* did not change significantly, so it is hard to conclude that the motility deficiency in the mutant had relationship with its flagella rotation (the function of the two *mot* genes, Table 2). In a NaCl-free medium the five genes showed a modest, but insignificant, increase in expression in the △wzx**_C1_** mutant strain compared to the wild-type strain. This indicates that the deficiency in *flgA* expression in the presence of NaCl may contribute to the observed salt-dependent decrease of motility.

## Discussion

The *wzx_C1_* gene, which is located in the O-antigen gene cluster, was identified in our previous work as being specific for *Salmonella* serogroup C1 [18]. In the present study, we provide experimental evidence to reveal the relationship between this gene and the O-polysaccharide biosynthesis of serogroup C1. We also found that the △wzx_C1_ mutant displayed NaCl-dependent motility deficiency, decreased flagellar expression and increased auto-aggregation.

Our results provide several lines of evidence that *wzx_C1_* encodes a putative flippase required for O-polysaccharide biosynthesis. Since structural knowledge of TM proteins is difficult to attain experimentally, the *in silico* prediction of membrane protein topology, such as the positions and orientation of the membrane-spanning regions, serves as a facile means to quickly obtain fundamental structural data of TM proteins. TOPCONS is a freely available web server for consensus prediction of membrane protein topology, and the topological prediction algorithms used in TOPCONS are sufficient in almost 70% of instances to correctly predict the overall number of putative TMs, as well as the orientation of the protein in the IM [32]. Wzx proteins share very little amino acid sequence similarity, and their genes also have low nucleotide sequence homology. A high level of variation in amino acid sequence identity of the flippases is observed between species and even among serogroups of the same species [33]. In this study, we also found that Wzx_C1_ shared limited deduced amino acid sequence identity with other Wzx flippases (BLASTp), however, the topological prediction algorithms revealed very high similarity between the Wzx_C1_ and Wzx from *S.* Typhimurium and *E. coli* O157:H7 (Fig. S1), whose topological profiles have been reported based on experimental data [21], [22]. It has been reported that O-antigen construction in *S.* Choleraesuis is made of repeating units of the identical mannose sugar (i.e., homopolymeric O antigens), while the O-antigen of *S.* Typhimurium or *E. coli* O157:H7 are heteropolymeric, being composed of various sugars such as mannose, rhamnose and galactose [13]. In fact, the topological predictions of *S.* Typhimurium and *E. coli* O157:H7 Wzx proteins exhibited more similarity to each other (32% sequence identity) than to the *S.* Choleraesuis Wzx_C1_, especially when using the ZPRED and △G predictions at position 200 to 300 (Fig. S1), which might be related to the difference in the O-antigens polysaccharide substrates for the respective flippases. Therefore, further topology mapping of Wzx_C1_ by use of randomly fused N/C-terminal reporter methodology as well as targeted deletion fusions will be of benefit to uncover its critical sites [34]. The LPS profiles obtained by SDS-PAGE analysis showed that all the bands of the O-antigen were missing in the LPS produced by the *wzx_C1_* mutant (Fig. 1A), an observation that is consistent with the results described by Liu *et al.* [35] in *S.* Typhimurium P9351 and Burrows and Lam [36] in *Pseudomonas aeruginosa*. Since O_7_ is present in all strains of the C1 serogroup, an O_7_ antibody agglutination test is the most commonly used method for serological characterization for identification of *Salmonella* serogroups C1. In this study, mutation of *wzx_C1_* resulted in negative reactions with the O_7_ slide agglutination test, while, positive reactions appeared in the wild-type and the complemented mutant strain (Fig. 1B). Our results demonstrated that *wzx_C1_* encodes a flippase required for synthesis of the O_7_ antigen common to strains within the C1 serogroup. Therefore, this *Salmonella* serogroup-specific gene was shown to be involved in O-antigen biosynthesis.

In this study, loss of O-antigen flippase in *S.* Choleraesuis resulted in significant defects in both swimming and swarming motilities (Figs. 2A and 2C). This result differs from the previous report in which deletion of the *S*. Typhimurium *wzx_ST_* had little effect on swimming motility [15]. In addition, the mutation of *wzx_C1_* resulted in an aggregative phenotype(Fig. 3C and Fig. 4B). This propensity was also observed in LPS synthesis defective mutants of *Citrobacter freundii* swarming on agar surfaces [37] and *Stenotrophomonas maltophilia*s swimming in TS broth [38]. Moreover, it is speculated from the TEM observations that deletion of *wzx_C1_* resulted in an observable reduction in flagellation, with most of the cells aggregating into bunches of three or more cells nearly devoid of flagella (Fig. 3C) with a smaller number of individual cells that appear only partially flagellated (Fig. 3D). Also, our results with the RT-qPCR assay (Fig. 6) demonstrated that reduced flagellar expression of genes involved in synthesis of flagella contributed to this swimming defect. Previous studies with *Campylobacter jejuni* have correlated the expression of flagella with auto-aggregation [39], [40]. In *E. coli*, the auto-aggregation mediated by aggregation protein Ag43 can interfere with motility in the presence of low levels of flagella [41]. Our results together with these reports show that auto-aggregation and motility are antagonistic phenotypes. It is likely that regulatory pathway/factors involved in flagellation balance between auto-aggregation and motility.

The loss of surface O-antigen prevented swarming because it normally acts to increase “wettability” by extracting water from the agar [15], thus the addition of a bio-surfactant can restore swarming motility. In this study, the swarming defect in the *wzx_C1_* mutant strain was rescued by the addition of bio-surfactant (Fig. 2E), which indicates that O-antigen also acted as a bio-surfactant, aiding in motility, in *S.* Choleraesuis. Flagellar upregulation was regarded as a marker for swarmer cell differentiation [17]. FlhDC is a well-characterized flagellar master regulator of *S. enterica*, and the up-regulation of *flhDC* expression is required for the high-level expression of flagella [42]. Nevertheless, neither the up-regulation of *flhDC* expression nor the down-regulation of *rcsB* (the response regulator of *flhDC*) was observed in this study (data not shown) during swarming, which was consistent with the report from Partridge and Harshey [43] that *Salmonella* cells do not upregulate flagellar gene expression to increase flagellar numbers during swarming.

In addition, we found that the removal of NaCl in the medium restored both swimming and swarming motilities of the *wzx_C1_* defective mutant (Figs. 2B and 2D). Moreover, auto-aggregation (Figs. 3F and 4B) and reduced flagellar gene expression (Fig. 6) were also relieved when *wzx_C1_* mutant cells were grown in the absence of NaCl. These recovery phenomena might be related with the alteration in osmotolreance to NaCl of the *wzx_C1_* mutant (Fig. 4A). Wzx flippases belong to the polysaccharide transporter (PST) family of proteins, which is classified as one of four members of the multidrug/oligosaccharidyl-lipid/polysaccharide (MOP) exporter superfamily. Recently, it has been observed that Wzx forms cationic lumen channel that mediates anionic O-antigen subunit translocation in *P. aeruginosa* [44], [45]. Another member of the MOP exporter superfamily, multidrug and toxin extrusion (MATE) proteins, have been extensively found to utilize Na^+^ as the coupling ion during extrusion of hydrophobic compounds from the cytoplasmic side via antiport [46]. Our recovery results following removal of the NaCl in the medium showed that this cationic lumen channel formed by Wzx_C1_ may be indicative of Na^+^-dependent transport. Furthermore, it has been reported that changes in external osmolality (achieved by varying NaCl concentration in growth media) regulate flagellar expression by the EnvZ pathway in *E. coli* [47] and the RcsB-RcsC signaling pathway in *S.* Typhi [48]. Therefore, further research is needed to reveal why a deletion of Wzx_C1_ flippase may mediate a NaCl-dependent motility deficiency, increased cell-cell aggregation, and changes in flagellation in *S.* Choleraesuis.

In conclusion, a *Salmonella* serogroup C1-specific gene, designated as *wzx_C1_,* encodes a putative flippase required for O_7_-polysaccharide biosynthesis. Auto-aggregation and a defect in flagellar expression were responsible for the reduced swimming motility in a *wzx_C1_* mutant strain. The O-antigens in *S.* Choleraesuis may act as a surfactant to regulate swarming motility. Motility deficiency, a cell aggregation phenomenon, and decreases in cell flagellation could be relieved by the removal of NaCl in the medium, which suggests that this serogroup C1-specific gene *wzx_C1_* is involved in salt tolerance and the Wzx_C1_ flippase probably displays Na^+^-dependent antiport activity.

## Supporting Information

Figure S1 Topology prediction profile of Wzx proteins. The topology prediction profile of the putative Wzx open reading frames from *S.* Choleraesuis (A), *S.* Typhimurium (B), and *E. coli* O157:H7 (C). The consensus prediction of these membrane protein topologies were generated by TOPCONS (http://topcons.cbr.su.se/).
(TIF)
