# Supplementary figures and images for: Mutation of a Salmonella Serogroup-C1-Specific Gene Abrogates O7-Antigen Biosynthesis and Triggers NaCl-Dependent Motility Deficiency
Source: PLoS One. 2014 Sep 11;9(9):e106708. doi: 10.1371/journal.pone.0106708 (PMC4161368; doi:10.1371/journal.pone.0106708)

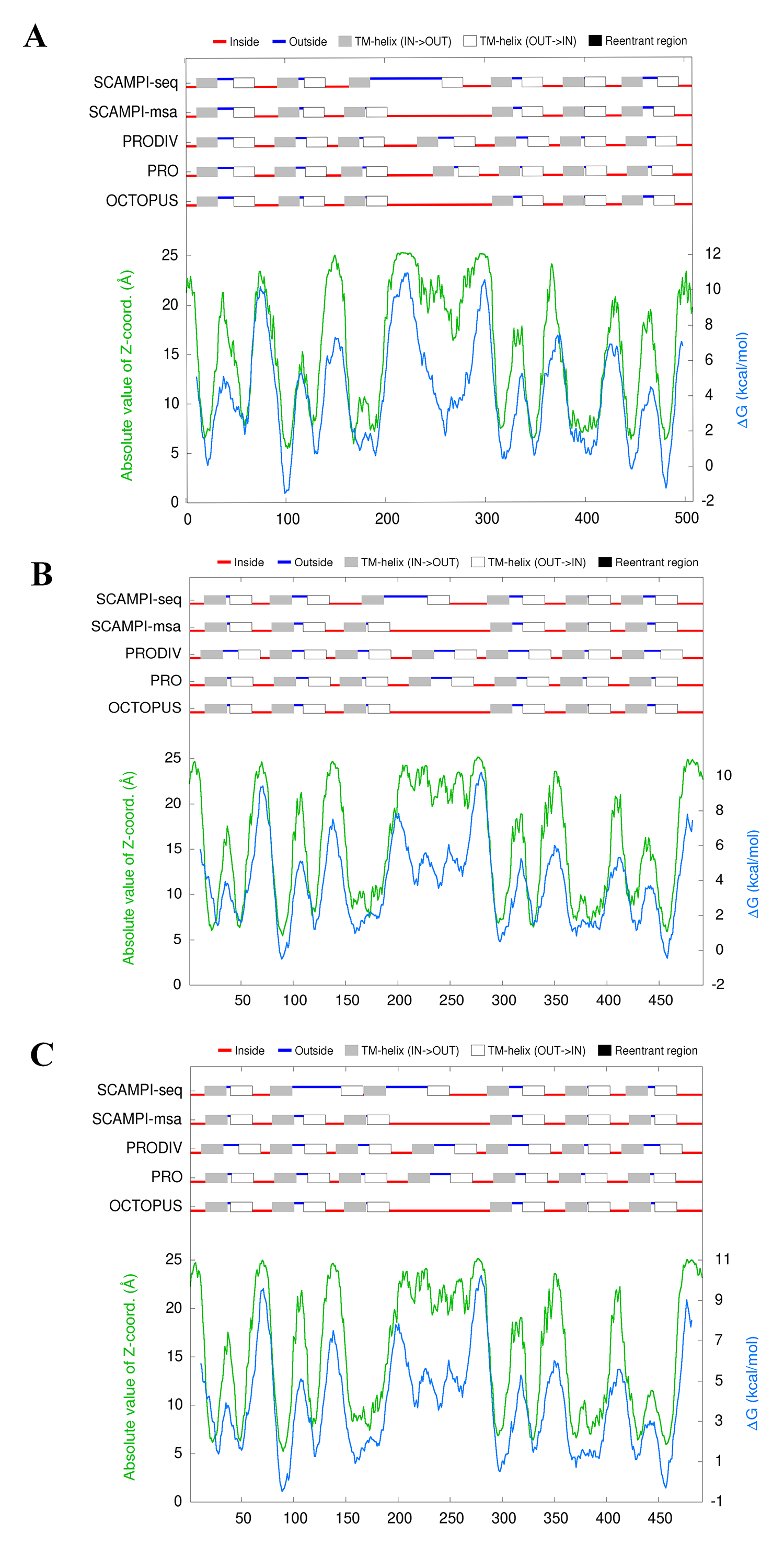

Supplement: Figure S1 — Topology prediction profile of Wzx proteins. The topology prediction profile of the putative Wzx open reading frames from S. Choleraesuis (A), S. Typhimurium (B), and E. coli O157:H7 (C). The consensus prediction of these membrane protein topologies were generated by TOPCONS (http://topcons.cbr.su.se/). (TIF) [file pone.0106708.s001.tif]
